# Identification of the miRNA signature associated with survival in patients with ovarian cancer

**DOI:** 10.18632/aging.202940

**Published:** 2021-04-27

**Authors:** Srinivasulu Yerukala Sathipati, Shinn-Ying Ho

**Affiliations:** 1 Center for Precision Medicine Research, Marshfield Clinic Research Institute, Marshfield, WI 54449, USA; 2 Institute of Bioinformatics and Systems Biology, National Chiao Tung University, Hsinchu, Taiwan; 3 Institute of Population Health Sciences, National Health Research Institutes, Miaoli, Taiwan; 4 Institute of Bioinformatics and Systems Biology, National Yang Ming Chiao Tung University, Hsinchu, Taiwan; 5 Department of Biological Science and Technology, National Yang Ming Chiao Tung University, Hsinchu, Taiwan; 6 Center For Intelligent Drug Systems and Smart Bio-devices (IDS2B), National Yang Ming Chiao Tung University, Hsinchu, Taiwan

**Keywords:** miRNA signature, survival estimation, ovarian cancer, machine learning

## Abstract

Ovarian cancer is a major gynaecological malignant tumor associated with a high mortality rate. Identifying survival-related variants may improve treatment and survival in patients with ovarian cancer. In this work, we proposed a support vector regression (SVR)-based method called OV-SURV, which is incorporated with an inheritable bi-objective combinatorial genetic algorithm for feature selection to identify a miRNA signature associated with survival in patients with ovarian cancer. There were 209 patients with miRNA expression profiles and survival information of ovarian cancer retrieved from The Cancer Genome Atlas database. OV-SURV achieved a mean correlation coefficient of 0.77±0.01and a mean absolute error of 0.69±0.02 years using 10-fold cross-validation. Analysis of the top ranked miRNAs revealed that the miRNAs, hsa-let-7f, hsa-miR-1237, hsa-miR-98, hsa-miR-933, and hsa-miR-889, were significantly associated with the survival in patients with ovarian cancer. Kyoto Encyclopedia of Genes and Genomes pathway analysis revealed that four of these miRNAs, hsa-miR-182, hsa-miR-34a, hsa-miR-342, and hsa-miR-1304, were highly enriched in fatty acid biosynthesis, and the five miRNAs, hsa-let-7f, hsa-miR-34a, hsa-miR-342, hsa-miR-1304, and hsa-miR-24, were highly enriched in fatty acid metabolism. The prediction model with the identified miRNA signature consisting of prognostic biomarkers can benefit therapeutic decision making of ovarian cancer.

## INTRODUCTION

Ovarian cancer is one of the deadliest types of gynaecological malignant tumors, resulting approximately 125,000 deaths worldwide each year [1]. Among ovarian cancer types, approximately 80–90% of cases are epithelial ovarian cancer. Some of the genetic risk factors for ovarian cancer include mutations in the tumor suppressor genes *BRCA1* and *BRCA2* [2]. Among patients with ovarian cancer, 8% have *BRCA1* mutation, 6% have *BRCA2* mutations, 6% have somatic *BRCA1/2* mutations, and 10% have *BRCA1* promoter inactivation [3]. Epithelial ovarian cancers spread to the adjacent organs first and later spreads to the liver and lungs, although bone and brain metastases are rarely observed. The survival time of patients with ovarian cancer varies at different stages; the 10-year survival rate is ~30% in all patients, and a recent study showed that after initial chemotherapy, the 5-year mortality rate was 65% [1, 4]. The treatment for ovarian cancer is determined by a combination of surgery and chemotherapy, either primary debulking surgery followed by chemotherapy or neo-adjuvant chemotherapy followed by interval debulking and chemotherapy [5]. Because of difficulties associated with early-stage detection and mass screening, ovarian cancer is the most lethal among all gynaecologic cancers.

MicroRNAs (miRNAs) are small noncoding RNAs that have attracted increasing attention owing to their potential involvement in initiation, progression, and metastasis [6] of various cancers. Several studies have shown that the expression of noncoding RNAs is associated with various types of cancer; hence, these RNAs have been used for predicting cancer types [7–10]. Moreover, miRNA and gene expression profiling have been used for prediction of survival and as effective molecular diagnostic markers in various cancers [2, 11, 12]. Recently, relationship between miRNA profiles and ovarian tumors has been studied extensively [13]; this study revealed that most miRNAs are downregulated in epithelial ovarian cancer and thus are associated with genomic copy number loss [14], indicating that miRNA expression is affected by genomic alterations. Studies on the dysregulation of miRNA expression in cancer may help to target oncogenes and to suppress their functions, thereby improving cancer treatment.

There are many studies that have discovered miRNA signatures for the survival analysis and prediction of progression in ovarian cancer. For instance, Shih et al. identified two miRNAs miR-410 and miR-645 that are negatively associated with the overall survival in advanced serous ovarian carcinoma [15]. Bagnoli et al. identified 35 miRNAs to create a prognostic model, and predicted risk of progression or relapse in ovarian cancer [16]. Besides the ovarian cancer, miRNA signatures have been used to predict survival in other cancer types, such as glioblastoma [17], lung cancer [18], and hepatocellular carcinoma [19]. Additionally, miRNA signatures have used to predict new subtypes of cancers. For instance, mRNA and miRNA expression signatures were used to predict subtype of serous ovarian cancer [20]. Bhattacharyya et al. used miRNA signatures for identifying subtypes of breast cancer [21]. Recently, various effective computational methods have been developed to understand the miRNA disease association [22, 23]. Hence, miRNA signatures are proven to be important factors for predicting survival and subtypes in cancers.

Various machine learning methods have been developed for cancer typing and diagnosis, including subtyping of liver cancer [24], breast cancer [25], lung cancer [26], and ovarian cancer [27]. These studies have used different machine learning models, such as support vector machines (SVMs) and artificial neural networks, along with various validation methods. Notably, SVMs have already been used to categorise the stages of ovarian cancer. De Smet et al. classified 31 patients with ovarian cancer into a stage I subgroup and an advanced-stage subgroup using least-square SVMs and principle component analysis, showing good accuracy [28]. Hartmann et al. used the cDNA gene expression profiles of 79 patients with ovarian carcinoma to predict early and late relapse [29]. Gevaert et al. have also estimated advanced-stage cisplatin resistance and stage I cisplatin sensitivity by means of microarray data obtained from 20 patients with ovarian cancer and observed a poor prediction [30]. Lisowska et al. utilised SVMs and Kaplan-Meier curves to predict disease-free survival and overall survival among 97 patients with ovarian cancer and observed no statistical difference in gene expression in the validation sets [31].

Only a few studies have therefore addressed prediction of cancer survival by means of a machine learning approach. One of the general issues with the use of machine learning methods is data quality, which is affected by noise and by missing and repetitive data. Thus, adequate data processing protocols are necessary for successful use of machine learning for the above purpose; feature selection plays a key role in reducing the number of unessential features to obtain a robust model. Overfitting is another general issue with the use of machine learning methods for predictive analysis of cancer. One of the known ways to avoid overfitting is cross-validation [32]. During cross-validation, a training dataset is used as a model and for testing. A comparison of model selection methods suggested that the 10-fold cross-validation (10-CV) method is the best approach for model selection [33].

Currently established treatments often fail to cure ovarian cancer. Substantial efforts have been made to find better therapeutic modalities to cure this cancer. As described above, to identify survival-related variants in ovarian cancer, miRNA expression data are often examined. Accordingly, the main aim of this work was to identify the miRNA signature that could estimate the survival of patients with ovarian cancer. In this work, we used The Cancer Genome Atlas (TCGA) database to retrieve miRNA expression profiles from 209 patients with ovarian cancer. We proposed a support vector regression (SVR)-based estimator (OV-SURV) for identification of miRNA signatures for the prediction of survival time in patients with ovarian cancer. As far as authors concern, this is the first study to use miRNA expression profiles and SVR to estimate the survival time in patients with ovarian cancer. OV-SURV identified a miRNA signature that associated with survival time of patients with ovarian cancer. We conducted 10-CV to assess the performance of OV-SURV. As a result, OV-SURV achieved a mean correlation coefficient of 0.77±0.01, with a mean absolute error of 0.69±0.02 years when comparing real and estimated survival time. Further, we seek to characterize the miRNA signature, as well as to understand the molecular mechanism in ovarian cancer survival by using bioinformatics approaches.

OV-SURV approach was based on an SVR and included an optimal feature selection method referred to as the inheritable bi-objective combinatorial genetic algorithm (IBCGA) [34]. Previously, an optimised SVR method was used to estimate the survival time of patients with glioblastoma [17], lung adenocarcinoma [18] and neuroblastoma [35]. This study could be ascribed to the following factors. Firstly, identifying the survival associated biomarkers in cancers are always necessary which could contribute to the prevention and diagnosis of cancers. Secondly, we customized the optimization method and tuned the parameters of OV-SURV, and prioritized the miRNAs of the signature to explore their roles in ovarian cancer. Thirdly, the prognostic power of the top ranked miRNAs in ovarian cancer was validated using survival curves. Fourthly, the biological significance of the identified miRNA signature was analyzed in terms of pathway analysis and functional annotations. Finally, importance of the top ranked miRNAs in ovarian cancer was verified by experimental literature. Further, we performed differential expression analysis and gene target prediction for the top ranked miRNAs. We hope that our findings can help identify new ovarian cancer-related miRNAs and contribute to the development of prognostic biomarkers in near future.

## Results and Discussion

### Identification of miRNA signature associated with survival time

First, we attempted to estimate the survival time of patients with ovarian cancer using miRNA expression profiles. We used a dataset containing 209 patients with ovarian cancer along with the expression profiles of 415 miRNA and survival time data. OV-SURV included the optimal feature selection algorithm IBCGA and identified a miRNA signature consisting of 39 miRNAs that could be used to predict the survival time of patients with ovarian cancer.

**Figure 1:**
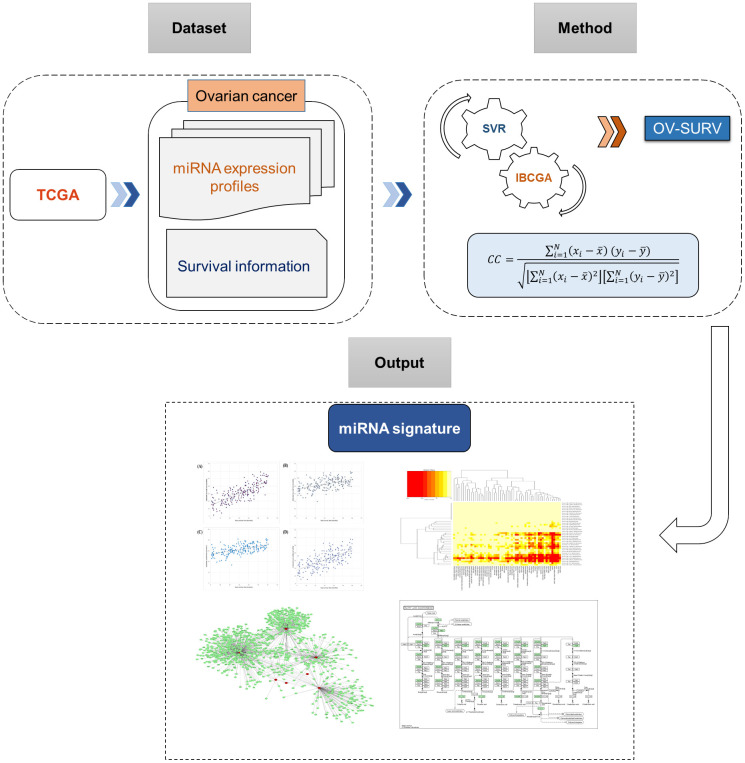
System flowchart of the current study describing the discovery of miRNA signature for predicting survival time in ovarian cancer.

The OV-SURV-average yielded a correlation coefficient of 0.77±0.01 and mean absolute error of 0.69±0.02 years between real and estimated survival time. The data strongly suggested that the identified miRNAs were effective at estimating the survival time of patients with ovarian cancer. The system flow chart of this work is depicted in Figure 1.

**Figure 2:**
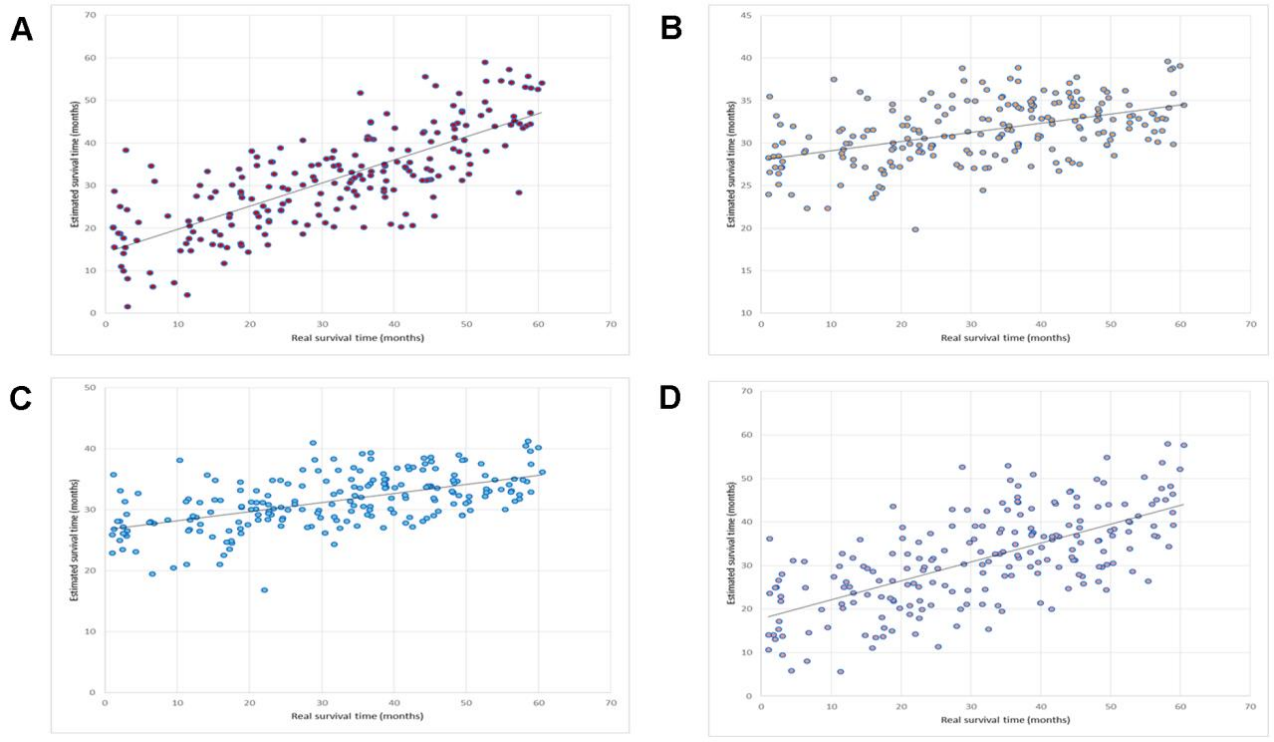
**Prediction of the performance of OV-SURV.** (**A**) OV-SURV achieved a correlation coefficient of 0.76. (**B**) LASSO yielded a correlation coefficient of 0.48. Real survival time in months is shown on the X-axis, and estimated survival time in months is shown on the Y-axis. (**C**) Elastic net obtained a correlation coefficient of 0.55. (**D**) Multiple linear regression obtained a correlation coefficient of 0.66. Real survival time in months is shown on the X-axis, and estimated survival time in months is shown on the Y-axis.

We compared this OV-SURV method with standard multiple linear regression [36], LASSO [37], and Elastic net [38]. The LASSO method resulted in a correlation coefficient and a mean absolute error of 0.48 and 1.00 years, respectively. The Elastic net method resulted in a correlation coefficient and a mean absolute error of 0.55 and 1.00 years, respectively. The multiple linear regression method resulted in a correlation coefficient and a mean absolute error of 0.66 and 0.84 years, respectively. A comparison of these results is shown in Table 1. The correlation plots between the estimated and real survival time obtained using OV-SURV, LASSO, Elastic net, and multiple linear regression are shown in Figure 2.

**Table 1:** Prediction of the performance of OV-SURV.

**Method**	**Identified** **miRNAs**	**10-CV correlation** **coefficient**	**Mean absolute** **error (years)**
LASSO	18	0.48	1.00
Elastic net	32	0.55	1.00
Multiple linear regression	18	0.66	0.84
OV-SURV	39	0.76	0.69
OV-SURV-mean	34.63	0.77±0.01	0.69±0.02

Further, OV-SURV was validated on an independent dataset from the TCGA database to evaluate the prediction performance. The dataset consisting of 160 living patients who suffer from ovarian cancer with follow-up time was used for the validation. The follow-up times of 160 patients were up to 5 years and the mean and standard deviation of the follow-up times were 20.42 ± 16.30 months. The mean estimated survival time of OV-SURV was 33.85 ± 5.24 months. There were 123 patients whose estimated survival time was longer than their actual follow-up time. The result revealed that OV-SURV achieved the classification accuracy of 77% for estimating the survival of patients with ovarian cancer. The mean absolute error of predicting the remaining 37 patients was 0.93 years which is slightly longer than the 0.69 years in Table 1. The prediction performance of OV-SURV on an independent test cohort was measured based on our previous study [18]. The prediction result of OV-SURV for each of the 160 patients is shown in Supplementary Figure 1.

### Ranking of the identified miRNA signature

To prioritize the miRNAs of the identified miRNA signature, miRNAs were ranked based on their contributions to the survival estimation using main effect difference (MED) analysis. There are 39 miRNAs in the identified miRNA signature. The 39 miRNAs of the signature, miRNA IDs, and MED scores are shown in Table 2. The top 10 miRNAs ranked by MED analysis were hsa-miR-19b, hsa-let-7f, hsa-miR-323, hsa-miR-1978, hsa-miR-128, hsa-miR-1237, hsa-miR-486, hsa-miR-98, hsa-miR-933, and hsa-miR-889, and these miRNAs were then analyzed further. Among the miRNAs in the signature identified by OV-SURV, these 10 miRNAs were significantly associated with survival time. We then assessed the involvement of these miRNAs and their corresponding genes in cancer and various biological pathways.

**Table 2:** Main effect difference (MED) scores of miRNA signatures.

**Rank**	**MiRNA**	**MED**
1	hsa-miR-19b	0.986621
2	hsa-let-7f	0.978907
3	hsa-miR-323	0.952427
4	hsa-miR-1978	0.867624
5	hsa-miR-128	0.821022
6	hsa-miR-1237	0.812224
7	hsa-miR-486	0.706714
8	hsa-miR-98	0.607252
9	hsa-miR-933	0.585211
10	hsa-miR-889	0.574037
11	hsa-miR-301b	0.568445
12	hsa-miR-514	0.561426
13	hsa-miR-935	0.530403
14	hsa-miR-653	0.494192
15	hsa-miR-1251	0.49296
16	hsa-miR-616	0.469875
17	hsa-miR-662	0.455425
18	hsa-miR-182	0.455374
19	hsa-miR-1245	0.435942
20	hsa-miR-200c	0.432309
21	hsa-miR-34a	0.36374
22	hsa-miR-187	0.353238
23	hsa-miR-190	0.343974
24	hsa-miR-342	0.329363
25	hsa-miR-513a	0.314319
26	hsa-miR-146b	0.306926
27	hsa-miR-1197	0.277773
28	hsa-miR-577	0.265914
29	hsa-miR-185	0.232879
30	hsa-miR-212	0.179705
31	hsa-miR-874	0.127656
32	hsa-miR-1304	0.122577
33	hsa-miR-106b	0.093269
34	hsa-miR-31	0.080119
35	hsa-miR-320d	0.04015
36	hsa-miR-1295	0.037462
37	hsa-miR-664	0.032954
38	hsa-let-7b	0.013824
39	hsa-miR-24	0.000766

### Validating the prognostic potential of top 10 ranked miRNAs

We validated the prognostic power of the top 10 ranked miRNAs using Kaplan-Meier (KM) survival curves. The TCGA dataset contains 486 tumor samples were considered for this analysis. Survival analysis was performed using Kaplan-Meir plotter [39]. Five of the top 10 ranked miRNAs, hsa-let-7f, hsa-miR-1237, hsa-miR-98, hsa-miR-933, and hsa-miR-889, were significantly associated with survival in patients with ovarian cancer. The survival groups were distributed using the log-rank test, a p-value<0.05 was considered the cut-off to describe the statistical significance in the survival analysis. These five miRNAs, hsa-let-7f, hsa-miR-1237, hsa-miR-98, hsa-miR-933, and hsa-miR-889, have p-values of 0.01, 0.00033, 0.007, 4.9e-0.5, and 0.011, respectively; and hazard ratios of 0.73, 0.61, 0.73, 1.62, and 1.4, respectively.

### Pathway analysis of the identified miRNA signature

The biological relevance of the selected miRNAs was analyzed using the DIANA tool. The pathway analysis helps to determine whether these miRNAs were statistically significant in Kyoto Encyclopaedia of Genes and Genomes (KEGG) pathways, including signalling pathways, cell processes and cancer pathways. The analysis revealed that the top 10 selected miRNAs were involved in several KEGG pathways, the most significant pathways including the transforming growth factor-β signalling pathway (hsa04350; p=3.29e-06), proteoglycans in cancer (hsa05205; p=7.93e-05), sphingolipid metabolism (hsa00600; p=0.0001), hippo signalling pathway (hsa04390; p=0.0005), and adherens junction (hsa04520; p=0.009). The details of the KEGG pathway analysis for the top 10 ranked miRNAs and their number of target genes are shown in Supplementary Table 1.

**Figure 3:**
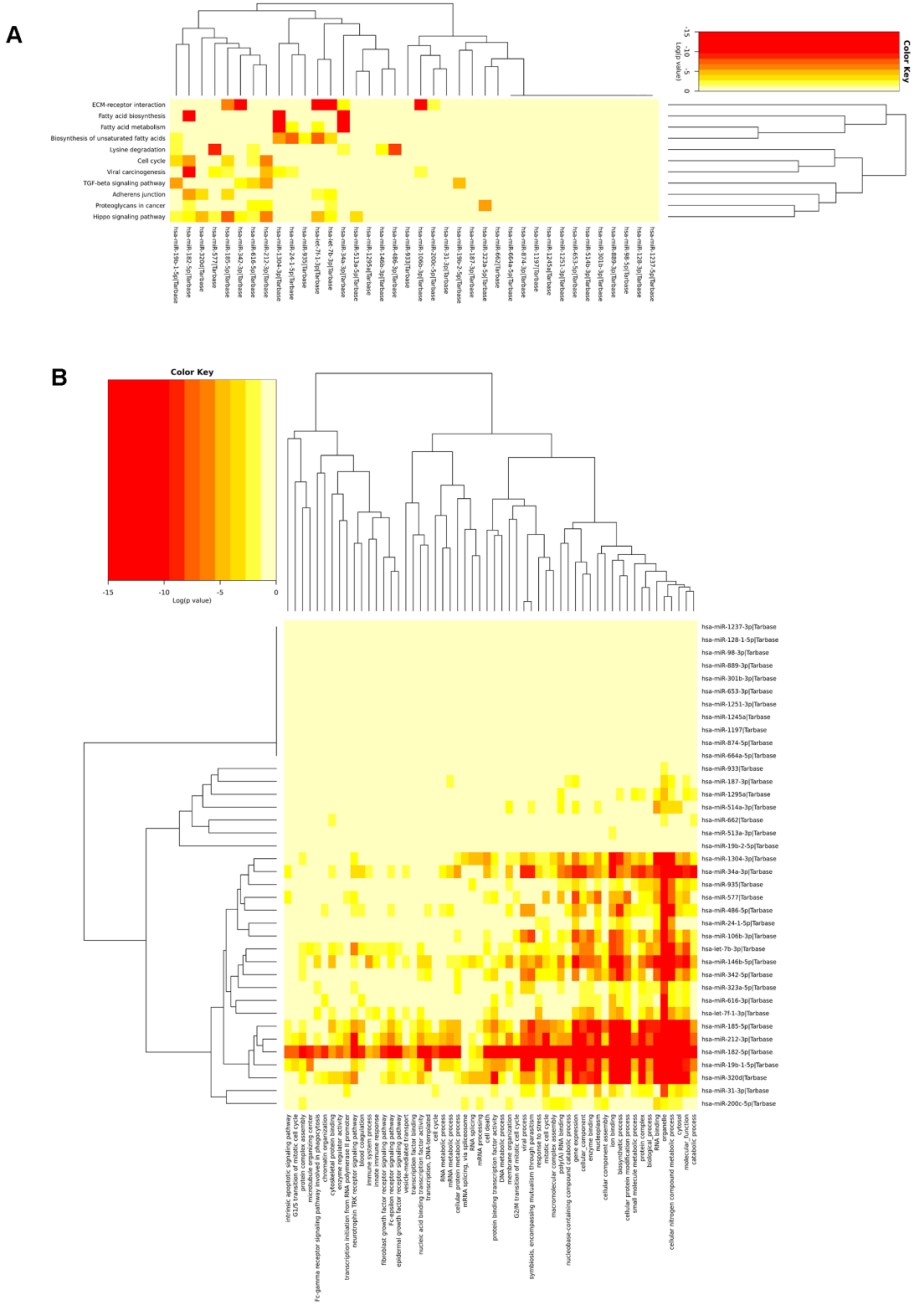
**Biological significance of the miRNA signature.** (**A**) miRNAs highly enriched in fatty acid metabolism, fatty acid biosynthesis, ECR receptor, and lysine degradation and (**B**) The miRNAs were found to be involved in different cellular, molecular, and biological pathways.

Additionally, the KEGG analysis of all 39 miRNAs in the signature revealed that few miRNAs were highly enriched in fatty acid biosynthesis, fatty acid metabolism, ECM/receptor interactions, and the hippo signalling pathway. The most highly significant pathway found in the KEGG pathway analysis was fatty acid biosynthesis (p<1E-325). There were four miRNAs (hsa-miR-182, hsa-miR-34a, hsa-miR-342, and hsa-miR-1304) that were highly enriched in fatty acid biosynthesis (hsa00061) and targeted genes such as *FASN*, *ACSL4*, and *ACACA.* Additionally, five miRNAs, namely, hsa-let-7f, hsa-miR-34a, hsa-miR-342, hsa-miR-1304, and hsa-miR-24, targeted the genes *FASN*, *PTPLB*, *SCD*, *ACSL4*, *ECHS1*, and *TECR*, which were all involved in fatty acid metabolism (p=1.02E-14). Fatty acid synthase (FAS) is an enzyme responsible for the synthesis of fatty acids and has been identified in most human carcinomas. FAS protein expression is associated with poor prognosis in both prostate and breast cancers [40, 41]. An *in vivo* microarray study revealed that there was a significant relationship between phosphorylated AKT and the expression of FAS; inhibition of FAS activity resulted in down regulation of phosphorylated AKT, which initiates apoptosis in ovarian cancer cells [42]. Inhibition of FAS and activation of AMP-activated protein kinase is selectively cytotoxic to SKOV3 human ovarian cancer cells [43]. Additionally, inhibition of FAS is significantly associated with survival in xenograft models [44]. This analysis suggested that the selected miRNAs were relevant to cancer and related biological signalling pathways. The heatmap showing KEGG pathway enrichment analysis of the miRNA signature is shown in Figure 3A.

Thus, this analysis indicated that fatty acid synthesis in ovarian cancer cells played an essential role in ovarian cancer progression. The identified miRNAs involved in fatty acid synthesis and fatty acid metabolism may possess important roles in ovarian cancer survival. The results of the KEGG pathway analysis for the miRNA signature and its target genes (various pathways, numbers of miRNAs involved, and the corresponding p-values) are shown in Supplementary Table 2. Fatty acid biosynthesis and fatty acid metabolism flow diagrams are shown in Supplementary Figures 2, 3.

### Functional annotations of the miRNA signature

Functional annotations of the identified miRNA signature were analyzed using GO enrichment analysis. GO annotation analysis revealed that the top 10 ranked miRNAs were involved in several biological processes (BPs), cellular component (CCs), and molecular functions (MFs). The top 10 ranked miRNAs were involved in several BPs, such as gene expression (GO:0010467; p<1e-325), biosynthetic process (GO:0009058; p<1e-325), cellular nitrogen compound metabolic process (GO:0044403; p<1e-325), symbiosis, encompassing mutualism through parasitism (GO:0044403; p=2.78e-14), and viral process (GO:0016032; p=3.14e-13) by targeting 82, 360, 419, 68, and 62 genes, respectively, to name a few.

The top 10 ranked miRNAs were involved in various CCs, includes organelle (GO:0043226; p<1e-325), cytosol (GO:0005829; p=4.46e-13), protein complex (GO:0043234; p=2.55e-11), nucleoplasm (GO:0005654; p=9.93e-11), and microtubule organizing center (GO:0005815; p=0.004). The top 10 ranked miRNs involved in MFs, such as ion binding (GO:0043167; p<1e-325), RNA binding (GO:0003723; p=2.22e-15), enzyme binding (GO:0019899; p=2.09e-08), poly(A) RNA binding (GO:0044822; p=1.16e-07), protein binding transcription factor activity (GO:0000988; p=4.34e-05), and nucleic acid binding transcription factor activity (GO:0001071;p=0.006). In GO annotation analysis, among the BPs most genes involved in cellular nitrogen compound metabolic process (419 genes), in CCs most genes involved in organelle (834 genes), and in MFs, most genes are involved in ion binding (447 genes). The details of top 10 ranked miRNAs involvement in GO annotations is listed in Supplementary Table 3.

Go enrichment analysis revealed that miRNA signature is highly enriched in gene expression, catabolic process, RNA binding, cytosol, cellular nitrogen compound metabolic process, viral process cellular protein modification process, organelles, and enzyme binding. GO analysis of the miRNA signature is shown in Figure 3B. The results of the GO analysis for the miRNA signature and its target genes are shown in Supplementary Table 4.

### Expression difference of the top ranked miRNAs across stages

Further, we wished to enquire about the relative expression levels of the top ranked miRNAs across different stages of ovarian cancer. We employed relative miRNA expression analysis across stages of ovarian cancer using UALCAN web portal [45]. The expression analysis results provide the significant expression difference of top 10 ranked miRNAs across different stages of patients with ovarian cancer. Of the top 10 ranked miRNAs, except the miRNA, hsa-miR-1978, remaining miRNAs are significantly expressed across stage 2, 3, and 4 of patients with ovarian cancer. Statistical significance of the relative miRNA expression across stages of ovarian cancer patients is provided in Supplementary Table 5. Box-whisker plot representation of relative expression difference across stages of ovarian cancer patients is given for the top ranked miRNAs in Supplementary Figure 4.

### Roles of the top ranked miRNAs in ovarian cancer

The roles of the top 10 ranked miRNAs in ovarian cancer were analyzed using experimentally validated literature.

*hsa-miR-19b*: A quantitative real-time PCR study involving ovarian cancer cells showed that hsa-miR-19b was significantly expressed between cancer and control group with a p-value of 0.035 [46]. Over expression of hsa-miR-19b facilitated the invasion and migration of ovarian cancer cells (CAOV-3) and serves as an oncogenic role in ovarian cancer progression [47].

*hsa-let-7f*: Zheng et al. identified lower expression levels of hsa-let-7f in ovarian cancer samples when compared to controls, further lower levels of hsa-let-7f associated with poor prognosis of patients with ovarian cancer [48]. Elevated expression level of let-7f was found in primary ovarian carcinomas and suggesting a role of this miRNA in tumor progression [49].

*hsa-miR-323*: *In silico* miRNA expression analysis in ovarian cancer reported that dysregulation of hsa-miR-323 in ovarian cancer cells [50]. Differential expression of miR-323 was observed in epithelial ovarian cancer cells and miR-323 was significantly down-regulated in epithelial ovarian cancer cell lines compared to ovarian surface epithelium cells [14].

*hsa-miR-128*: hsa-miR-128 downregulates the colony stimulating factor-1, a key regulator of ovarian cancer, resulted into reduction of cell motility and adhesion in ovarian cancer cells [51].

*hsa-miR-486*: hsa-miR-486 downregulated the estrogen receptor alpha (ERα)-mediated olfactomedin 4, which contributes to ovarian cancer progression in cancer cells [52].

*hsa-miR-98*: A qRT-PCR study epithelial ovarian cancer revealed that hsa-miR-98 regulates the cisplatin resistance of epithelial ovarian cancer cells and was associated with poor outcome of patients with ovarian cancer [53].

A summary of the top 10 ranked miRNAs and their roles in ovarian cancer are shown in Table 3. In summary, six miRNAs, namely, hsa-miR-19b, hsa-let-7f, hsa-miR-323, hsa-miR-128, hsa-miR-486, and hsa-miR-98, had experimentally validated support to confirm their associations in ovarian cancer. The remaining four miRNAs, such as hsa-miR-1978, hsa-miR-1237, hsa-miR-933, and hsa-miR-889, were not previously reported to have roles in ovarian cancer. However, they were actively involved in other types of cancer, such as kidney cancer [54], breast cancer [55], gastric cancer [56], and squamous cell carcinoma [57]. Additionally, we verified the importance of these four miRNAs, hsa-miR-1978, hsa-miR-1237, hsa-miR-933, and hsa-miR-889 in ovarian cancer by performing overall survival analysis using Kaplan-Meir survival curves. The miRNAs, hsa-miR-1978, hsa-miR-1237, hsa-miR-933, and hsa-miR-889 obtained p-values of 0.22, 0.01, <0.001, and <001, respectively. The three miRNAs, hsa-miR-1237, hsa-miR-933, and hsa-miR-889 were significantly associated with the overall survival in patients with ovarian cancer. The KM survival plots for hsa-miR-889, hsa-miR-1237, and hsa-miR-933 are shown in Supplementary Figure 5.

**Table 3:** Summary of top 10 ranked miRNAs involved in ovarian cancer.

**Rank**	**miRNA**	**Cancer**	**Regulation**	**Source**
1	hsa-miR-19b	ovarian cancer	up	[66]
2	hsa-let-7f	ovarian cancer	down	[49]
3	hsa-miR-323	ovarian cancer	down	[14]
4	hsa-miR-1978	-	-	-
5	hsa-miR-128	ovarian cancer	down	[51]
6	hsa-miR-1237	-	-	-
7	hsa-miR-486	ovarian cancer	down	[52]
8	hsa-miR-98	ovarian cancer	down	[53]
9	hsa-miR-933	-	-	-
10	hsa-miR-889	-	-	-

Furthermore, we validated the involvement of top 10 ranked miRNAs in various cancers including ovarian cancer using experimentally validated studies. All of the top 10 ranked miRNAs were involved in major types of cancers, in which 6 miRNAs, hsa-miR-19b, hsa-let-7f, hsa-miR-323, hsa-miR-128, hsa-miR-486, and hsa-miR-98 were involved in ovarian cancer progression. The top 10 ranked miRNAs and experimentally validated evidences across various cancers is listed in Supplementary Table 6.

### Differential expression of miRNA signature in ovarian cancer

Furthermore, to examine the involvement of the miRNA signature and the expression levels of the miRNAs of the signature in ovarian cancer, we predicted the association of the miRNA signature with ovarian cancer using dbDEMC 2.0 and HMDD v3.0. The prediction results showed that 29 miRNAs were differentially expressed in ovarian cancer. The expression levels of the remaining 10 miRNAs had not been previously reported in ovarian cancer, although these miRNAs were known to be actively involved in other major cancer types. These results suggested that these 10 miRNAs, including hsa-miR-323, hsa-miR-1978, hsa-miR-933, hsa-miR-616, hsa-miR-1245, hsa-miR-19b-2, hsa-miR-342, hsa-miR-513a, hsa-miR-577, and hsa-miR-1295, should be further evaluated to determine their roles in ovarian cancer survival. The prediction list of the identified miRNA signature associated with ovarian cancer is shown in Supplementary Table 7.

Additionally, we predicted the miRNA gene target network for the top ranked miRNAs using Cytoscape version 3.6. There are total 5009 predicted gene targets, in which 3893 interactions are predicted using MicroCosm version 5, and 116 interactions are predicted using TargetScan version 6.2. The miRNA-gene target interaction network is visualized in Supplementary Figure 6, for better visualization, only gene targets predicted by TargetScan are shown.

## CONCLUSIONS

Considering experimental limitations and rapidly growing biological datasets, machine learning models are becoming necessary for the identification of cancer-related miRNAs, e.g., in cancer diagnosis. MiRNA profiling is now extensively used in cancer research and cancer treatment. Recently, various miRNAs have been shown to play important roles in ovarian cancer invasion and metastasis. Some miRNAs were significantly expressed in cancer tissues when compared to the normal tissues. For instance, miR-199a, and miR-200 were significantly expressed in ovarian cancer tissues when compared to the normal tissues [58]. There are some miRNAs which have been identified as prognostic biomarkers in ovarian cancer. For example, miR-1183, miR-126, miR-802, and miR-139 showed significant association with prognosis [59]. Bagnoli et al. identified miRNAs to predict early relapse/progression of ovarian cancer patients [16]. However, there are few studies using machine learning with optimization techniques to estimate the survival time of patients with ovarian cancer. Hence, in this work, we proposed a survival estimation method, called OV-SURV, that identified an miRNA signature associated with survival in patients with ovarian cancer. Machine learning methods for analysis of cancer survival are often faced the problem of overfitting. Accordingly, we addressed this issue in our OV-SURV model, which contained an optimal feature selection algorithm. OV-SURV yielded promising results and selected an informative miRNA signature that was associated with survival in patients with ovarian cancer. MED ranking revealed the 10 miRNAs that were the most significant with respect to cancer survival. Validation of top ranked miRNAs using KM survival analysis revealed the prognostic power of the miRNAs, of top 10 ranked miRNAs, five miRNAs were significantly associated with survival in patients with ovarian cancer. Moreover, among the identified miRNA signature, the finding of the four miRNAs hsa-miR-486, hsa-miR-514, hsa-miR-200c, and hsa-miR-513a was consistent to those in the study [16] for the association with the prognosis in patients with ovarian cancer. The identified miRNAs have prognostic value in ovarian cancer.

The biological significance of these miRNAs was analyzed by KEGG pathway and GO annotations, which indicated that the selected miRNAs were significantly involved in the regulation of fatty acid biosynthesis, fatty acid metabolism, and several other relevant signalling and cancer pathways. Identified miRNAs are significantly expressed across different stages of ovarian cancer. These expression differences of miRNAs might also affect the survival in patients with ovarian cancer. Further, the importance of top ranked miRNAs in ovarian cancer is discussed. Six of the top 10 ranked miRNAs role in ovarian cancer was verified by experimental validated literature. This analysis revealed that remaining four miRNAs of the top 10 ranked miRNAs, such as hsa-miR-1978, hsa-miR-1237, hsa-miR-933, and hsa-miR-889, were not previously reported to have roles in ovarian cancer. However, their contribution to the survival estimation revealed that these are the important subjects to explore further in ovarian cancer. Differential expression analysis of miRNA signature shown 29 of 39 miRNAs of the signature were significantly expressed in ovarian cancer cells. The miRNA-target network was constructed to visualize the target interactions of the top ranked miRNAs, derived from interaction databases.

The above findings showed the ability of the OV-SURV method to identify miRNA signatures that could predict survival in patients with ovarian cancer. Although the prediction performance of OV-SURV is promising, this method may be further improved by increasing the data size. Nonetheless, our model performed well compared with other machine learning methods in terms of 10-CV. The identified miRNAs were proven here to be relevant to ovarian cancer and other cancers. Because of increasing cancer-related mortality, computational predictive methods are necessary for the rapid identification of potential biomarkers. We believe that the miRNAs identified here will help to develop new prognostic or diagnostic biomarkers for ovarian cancer.

## MATERIALS AND METHODS

### Dataset

We obtained miRNA expression data from patients with ovarian cancer using the TCGA database. The number of patients with ovarian cancer in the TCGA database was 586. We downloaded level-3 miRNA expression data from the TCGA portal, and the miRNA profiling was performed using the Illumina HiSeq 2000 miRNA sequencing platform. We filtered the dataset using the following criteria: (i) only patients with clinical data and survival information were included; (ii) to statistically remove the bias, the patients with a survival period of less than 30 days were excluded; and (iii) all patient lists and the corresponding survival periods were merged into a single data file to eliminate duplicate entries. After this filtering process, we identified 209 patients with the expression profiles of 415 miRNAs along with corresponding clinical data and days to death. Additionally, we used the dataset consisting of 160 living patients who suffer from ovarian cancer with follow-up time for the validation. Clinical information of the independent test cohort is listed in Supplementary Table 8.

### OV-SURV

The proposed method OV-SURV was designed to estimate the survival time of patients with ovarian cancer by identifying a miRNA signature and then to discover their potential roles of miRNA biomarkers in ovarian cancer. SVM is effective for solving classification and regression problems [60] and can interpret the property values of a large number of samples in a multidimensional space. Owing to its effective regression abilities, SVR has been used to solve many biological prediction problems. The optimisation problem of ν-SVR for finding a model *w* can be defined as follows:

$$\min\;[\{\frac{1}{2}\;w^{T}(\emptyset (x_{i})+b)+C(\nu \varepsilon +\frac{1}{m}\sum_{i=1}^{m}\left(\xi_{i}+\xi_{i}^{*})\right)\}]$$ (1)

Here, 0 ≤ ν ≤ 1, *ξ_i_* ≥ 0, (***x****_1_*, *y_1_*)…(***x****_m_*, *y_m_*) F0CE *R^d^* ×*R* are training data, *y*_i_ is the target values, ***x****_i_* is the feature vector. We used *d* is the number of features in each instance and *m* is the number of training instance where *d*=415 and *m*=209 in this study. Let *C*>0 be the regularisation parameter, ε≥0 be an error sensitivity parameter, and *b* is a constant. The parameters *C* and ε are used to avoid overfitting the training data. The parameters *C*, ε, a Gaussian kernel γ and the feature selection are simultaneously optimized by OV-SURV while maximizing the estimation accuracy.

### IBCGA

The feature selection algorithm IBCGA uses an intelligent evolutionary algorithm (IEA) [34], which can efficiently solve large parameter optimisation problems, and is good at deriving an optimised SVR model with feature selection. IBCGA can automatically select a small set of informative features from a large number of candidate features. OV-SURV identifies a signature of informative miRNAs associated with ovarian cancer survival based on the cooperation of IBCGA and ν-SVR using the objective function of maximising the correlation coefficient (CC) in terms of 10-CV.

$$CC=\frac{\sum_{i=1}^{N}(x_{i}-\bar{x})\;(z_{i}-\bar{z})}{\sqrt{\left[\sum_{i=1}^{N}{\left(x_{i}-\bar{x}\right)}^{2}\right]\left[\sum_{i=1}^{N}{\left(z_{i}-\bar{z}\right)}^{2}\right]}}$$ (2)

where *x*_i_ and *z*_i_ are real and predicted survival time of the i-th miRNA, $\bar{x}$ and $\bar{z}$ are the corresponding means, and *N* is the total number of instances. Mean absolute error (MAE) is also used for estimating the prediction performance. The MAE is defined as follows

$$MAE=\;\frac{1}{N}\overset{N}{\underset{i=1}{\sum }}{\left|x-z\right|}^{2}$$ (3)

In this work, the LibSVM package [61] was used for implementation of ν-SVR.

The chromosome of IEA comprises 415 binary genes representing miRNAs for feature selection and three 4-bit genes for encoding parameters γ, C, and ν of ν-SVR. The chromosome encoding was designed as described in previous studies [17, 34]. The IBCGA can simultaneously obtain a set of solutions, *Xr*, where *r* = *r_end_, r_end +_* 1,*…*, *r_start_* in a single run. The feature selection algorithm IBCGA used can be described as follows:

Step 1: (Initialisation) Randomly generate an initial population of *Npop* individuals. In this work, *Npop*=50, *r_start_* = 50, *r_end_* = 10, *r* = *r_start_*.

Step 2: (Evaluation) Evaluate the fitness value of all individuals using the fitness function (CC) of 10-CV.

Step 3: (Selection) Use a conventional method of tournament selection that selects a winner from two randomly selected individuals to generate a mating pool.

Step 4: (Crossover) Select two parents from the mating pool to perform an orthogonal array crossover operation of IEA.

Step 5: (Mutation) Apply a conventional bit mutation operator to parameter genes and a swap mutation to the binary genes for keeping *r* selected features. The best individual was not mutated for the elite strategy.

Step 6: (Termination test) If the stopping condition for obtaining the solution *Xr* is satisfied, output the best individual as the solution *Xr*. Otherwise, go to Step 2.

Step 7: (Inheritance) If *r* > *r_end__,_* randomly change one bit in the binary genes for each individual from 1 to 0; decrease the number *r* by one and go to Step 2. Otherwise, stop the algorithm.

Step 8: (Output) Obtain a set of *m* miRNAs from the chromosome of the best solution *Xm* among the solutions *Xr*, where *r* = *r_end_, r_end +_* 1,*…*, *r_start_*.

### Multiple linear regression analysis

We employed multiple regression to estimate the survival time in patients with ovarian cancer [36]. A general multiple linear regression can be defined as

$$y_{i}=\beta_{0}+\beta_{1}x_{1}+\beta_{2}x_{2}+\cdots +\beta_{n}x_{n}+\varepsilon$$ (4)

### KEGG pathway and GO term analysis

We used the DIANA-mirPath version 3 web-based server to analyze the miRNA profiles [62]. The DIANA-micro-T-CDS algorithm provided the predicted miRNA targets for the pathway analysis. The p-value threshold was set to 0.05 and Fishers’s exact test (hypergeometric distribution) was used for the enrichment analysis. We utilised the Network of Cancer Genes (NCG) [63], miRTarBase [64], and GeneCards [65] databases to obtain the cancer-related genes and predict gene targets.

### Availability of data and materials

All the data used in this analysis can be found at TCGA data portal [https://cancergenome.nih.gov/].

## Supplementary Material

Supplementary Figures

Supplementary Tables
