# Supplementary material for: Identification of the miRNA signature associated with survival in patients with ovarian cancer
Source: Aging (Albany NY). 2021 Apr 27;13(9):12660–90. doi: 10.18632/aging.202940 (PMC8148489; doi:10.18632/aging.202940)
Supplement: Supplementary Figures [file aging-13-202940-s001.pdf]

SUPPLEMENTARY FIGURES

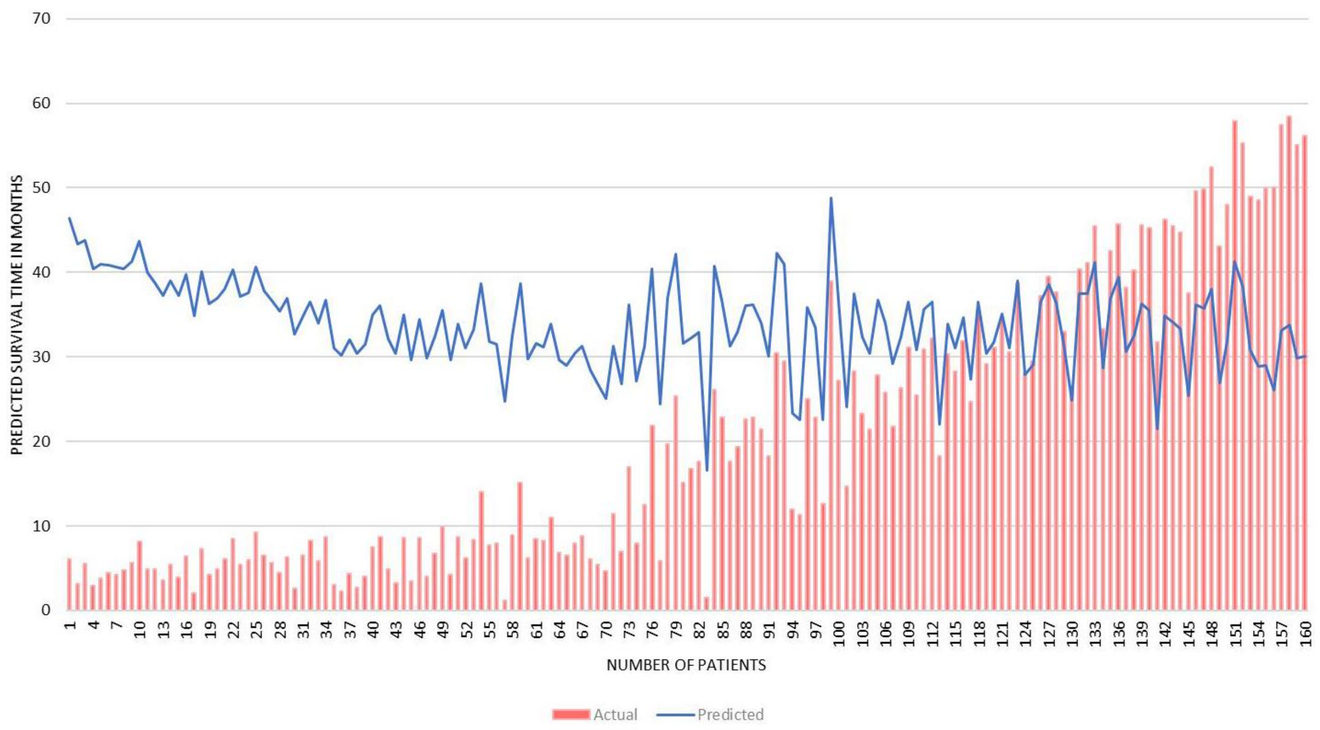

Supplementary Figure 1. The validation of OV-SURV on an independent cohort of 160 living patients with ovarian cancer. Predicted survival time is longer than the follow-up time for the first 123 patients (1–123).

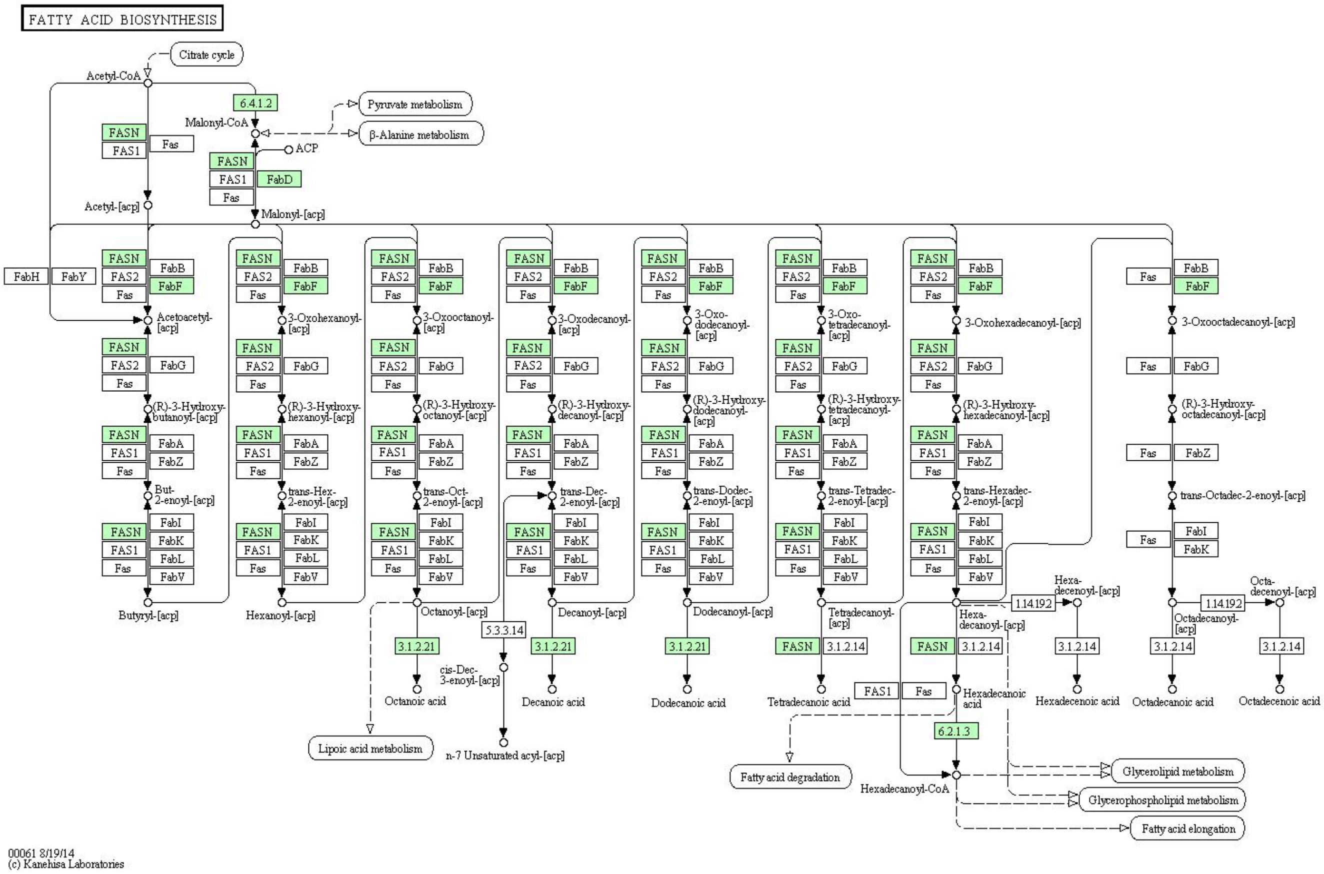

Supplementary Figure 2. Fatty acid biosynthesis flow diagram. Obtained from the KEGG pathway database.

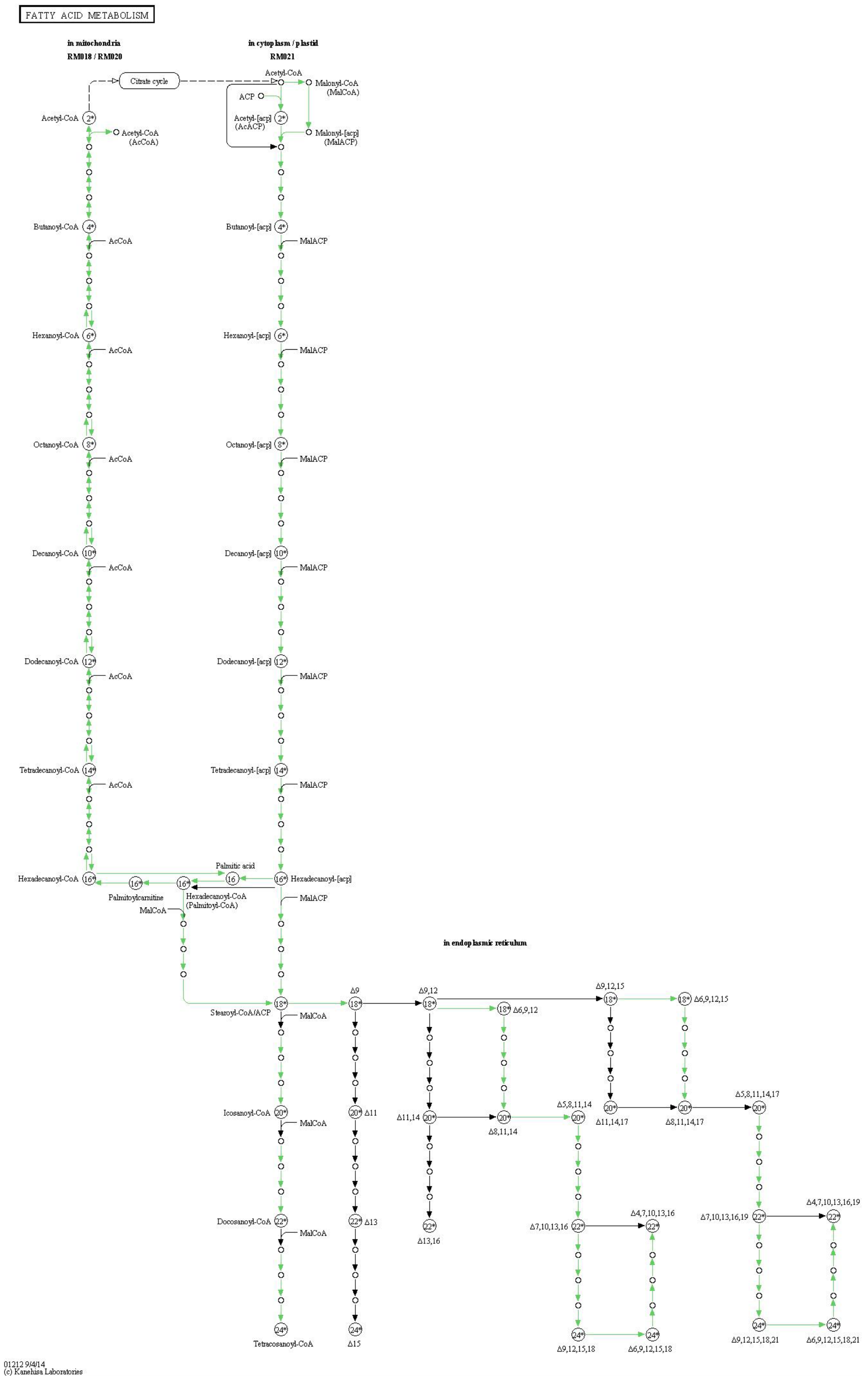

Supplementary Figure 3. Fatty acid metabolism flow diagram. Obtained from the KEGG pathway database.

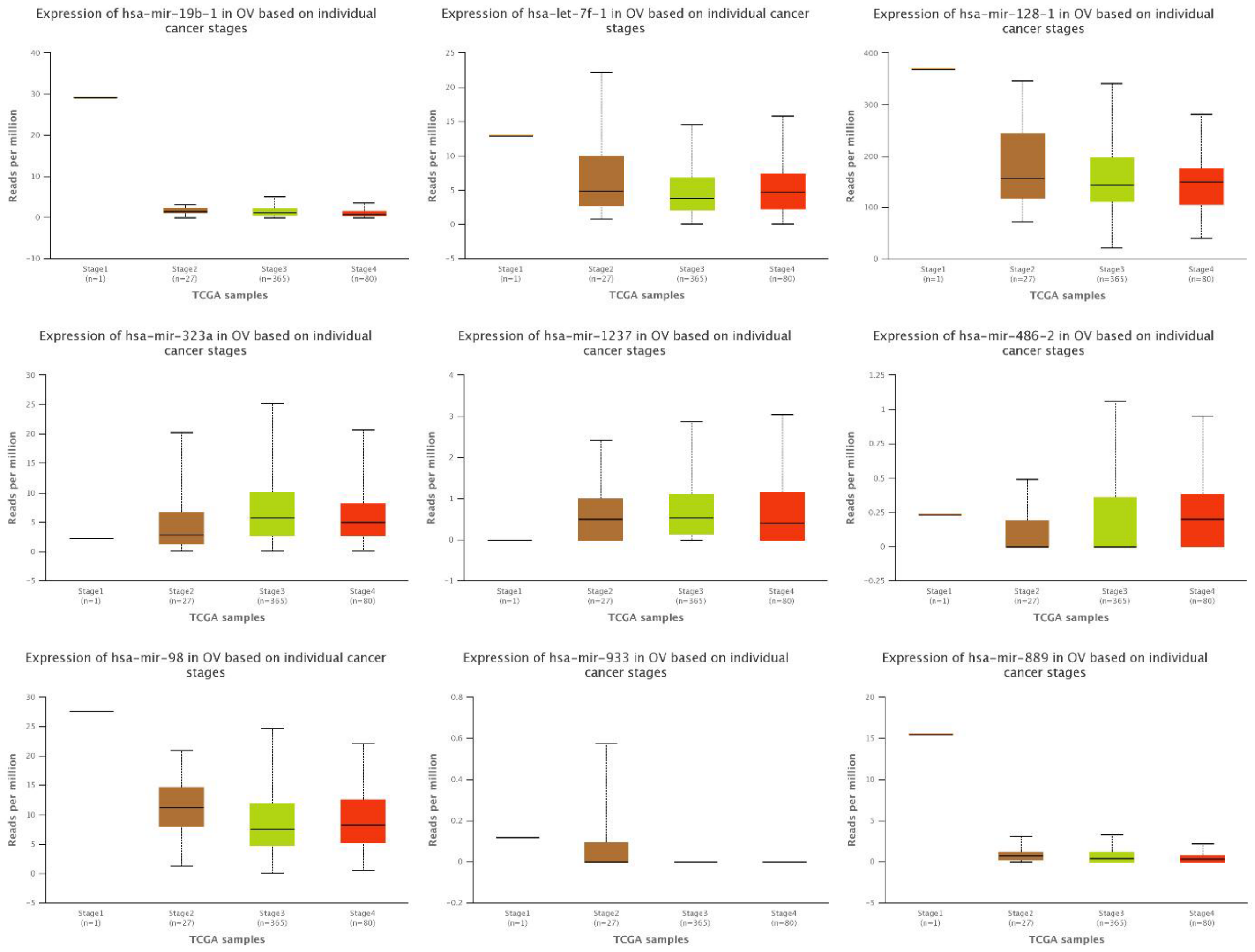

Supplementary Figure 4. Box plot showing relative expression of top ranked miRNAs across stages of patients with ovarian cancer.

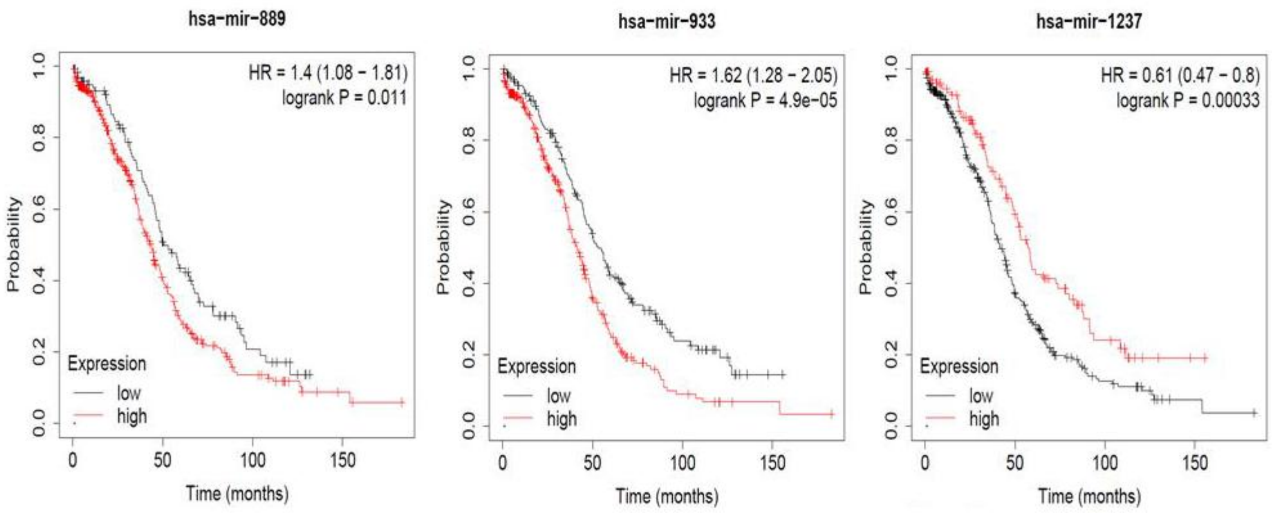

Supplementary Figure 5. Kaplan–Meier plots of hsa-miR-889, hsa-miR-933, and hsa-miR-1237 for the high-expression and low-expression groups of the ovarian cancer cohort.

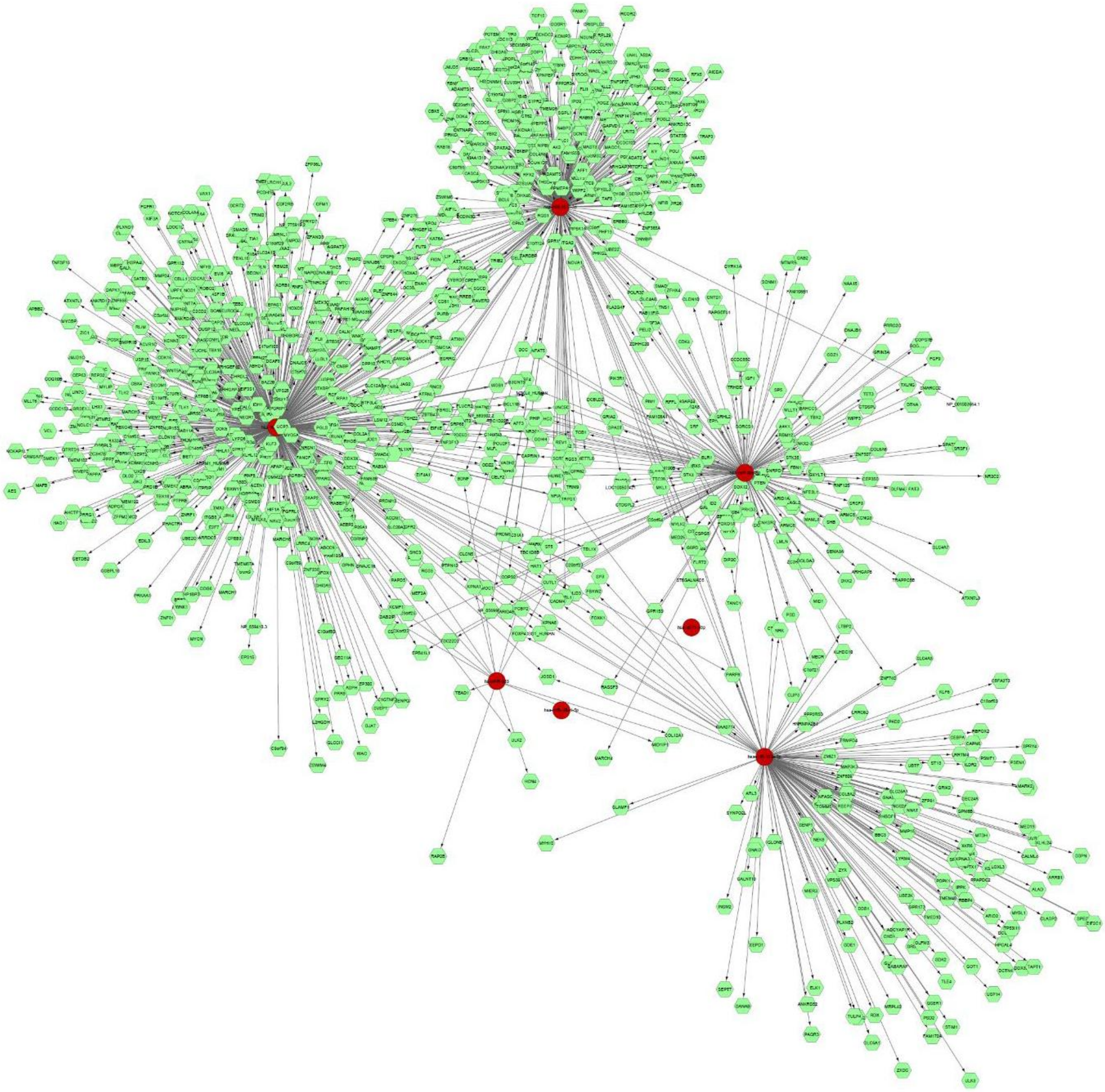

Supplementary Figure 6. miRNA-gene target network. miRNAs are shown in red colored circles; edges represent target genes in green colored hexagons. There are 1116 predicted interactions in the network using TargetScan.
