# Supplementary material for: Identification of the miRNA signature associated with survival in patients with ovarian cancer
Source: Aging (Albany NY). 2021 Apr 27;13(9):12660–90. doi: 10.18632/aging.202940 (PMC8148489; doi:10.18632/aging.202940)
Supplement: Supplementary Tables [file aging-13-202940-s002.pdf]

SUPPLEMENTARY TABLES

Supplementary Table 1. Top 10 ranked miRNAs involved in KEGG pathways.

KEGG pathway	No. of genes	no. of miRNAs	p-value
TGF-beta signaling pathway	16	5	3.289E-06
Proteoglycans in cancer	26	4	7.935E-05
Sphingolipid metabolism	10	3	0.0001055
Hippo signaling pathway	24	4	0.0005239
Adherens junction	13	5	0.0094746
Signaling pathways regulating pluripotency of stem cells	21	5	0.0106464
Lysine degradation	7	3	0.0191976
Cell cycle	18	5	0.0261571
Endocytosis	24	5	0.0261571
Regulation of actin cytoskeleton	26	5	0.0261571
ECM-receptor interaction	11	5	0.0315672
Glioma	11	4	0.0419673
FoxO signaling pathway	20	4	0.0498515

Supplementary Table 2. KEGG pathway analysis of the miRNA signature.

KEGG pathway	Genes	miRNAs	p-value
TGF-beta signaling pathway	57	26	2.553E-11
Axon guidance	76	26	5.697E-07
Hippo signaling pathway	87	27	8.597E-07
Renal cell carcinoma	46	26	1.388E-06
Signaling pathways regulating pluripotency of stem cells	87	30	2.115E-06
Adherens junction	46	20	4.679E-06
Proteoglycans in cancer	108	31	5.515E-06
Fatty acid metabolism	22	18	1.541E-05
Prion diseases	14	17	2.916E-05
Arrhythmogenic right ventricular cardiomyopathy (ARVC)	43	24	6.834E-05
Thyroid hormone signaling pathway	66	27	0.0004487
Pathways in cancer	212	35	0.0004487
Rap1 signaling pathway	113	30	0.0007618
Estrogen signaling pathway	54	26	0.0013402
Fatty acid biosynthesis	5	8	0.0017506
Lysine degradation	27	27	0.0019085
Glutamatergic synapse	62	31	0.0031419
Bacterial invasion of epithelial cells	45	28	0.0034732
FoxO signaling pathway	74	32	0.0053876
Circadian rhythm	23	27	0.0058225
Transcriptional misregulation in cancer	93	32	0.0058225
Focal adhesion	110	32	0.0073101
Dorso-ventral axis formation	20	18	0.0075923
Mucin type O-Glycan biosynthesis	16	15	0.0090565
Endocytosis	110	30	0.0093516

Oxytocin signaling pathway	83	31	0.0125495
Prostate cancer	51	31	0.0144126
Fatty acid degradation	20	15	0.0146739
Wnt signaling pathway	72	30	0.0146739
Platelet activation	71	30	0.0148846
Shigellosis	38	20	0.0162063
mRNA surveillance pathway	50	23	0.0162063
Thyroid hormone synthesis	36	26	0.0162063
Cell cycle	64	27	0.0162063
Neurotrophin signaling pathway	65	31	0.0162063
cGMP-PKG signaling pathway	86	34	0.0162063
Morphine addiction	44	26	0.0188883
Ubiquitin mediated proteolysis	70	28	0.0253401
Adrenergic signaling in cardiomyocytes	74	30	0.026999
Chronic myeloid leukemia	41	25	0.0277362
Regulation of actin cytoskeleton	108	31	0.0277362
Vasopressin-regulated water reabsorption	26	24	0.028921
T cell receptor signaling pathway	56	24	0.0352566
Glioma	35	28	0.0352566
Phosphatidylinositol signaling system	43	29	0.0352566
Protein processing in endoplasmic reticulum	83	32	0.0354841
Prolactin signaling pathway	39	24	0.0360174
GABAergic synapse	42	27	0.0360174
PI3K-Akt signaling pathway	167	35	0.0360174
RNA transport	85	31	0.0375067
Pancreatic cancer	37	27	0.041276
Melanoma	40	29	0.0430789
cAMP signaling pathway	100	34	0.0455181
Choline metabolism in cancer	55	28	0.0488212

Supplementary Table 3. GO analysis of top 10 ranked miRNAs involved in biological process, cellular components and molecular functions (p<0.05).

	GO category	miRNAs	Genes	p-value
Biological process	gene expression	3	82	1e-325
	biosynthetic process	4	360	1e-325
	cellular nitrogen compound metabolic process	4	419	1e-325
	symbiosis, encompassing mutualism through parasitism	3	68	2.787E-14
	viral process	3	62	3.15E-13
	cellular protein modification process	4	209	8.606E-13
	transcription, DNA-templated	2	170	3.92E-06
	catabolic process	2	131	1.38E-05
	response to stress	2	129	0.0002111
	nucleobase-containing compound catabolic process	2	71	0.0003728
	membrane organization	3	55	0.0005988
	small molecule metabolic process	2	138	0.0008035
	mitotic cell cycle	1	27	0.0017244
	cell death	2	62	0.0026435
	Fc-epsilon receptor signaling pathway	3	23	0.0033008
	neurotrophin TRK receptor signaling pathway	4	32	0.0034551
	mRNA metabolic process	2	23	0.0045806
	cellular component assembly	1	64	0.0061575
	DNA metabolic process	1	47	0.0103647
	macromolecular complex assembly	1	47	0.0109222
	viral life cycle	2	16	0.0129594
	RNA metabolic process	2	24	0.023756
	cellular protein metabolic process	2	31	0.0397643
Cellular components	organelle	5	834	<1e-325
	cytosol	4	248	4.462E-13
	protein complex	4	308	2.554E-11
	nucleoplasm	3	118	9.938E-11
	microtubule organizing center	1	36	0.004196
Molecular functions	ion binding	4	447	<1e-325
	RNA binding	4	192	2.22E-15
	enzyme binding	3	109	2.094E-08
	poly(A) RNA binding	2	124	1.168E-07
	protein binding transcription factor activity	2	46	4.346E-05
	nucleic acid binding transcription factor activity	2	63	0.0060971

Supplementary Table 4. GO analysis of the miRNA signature.

GO category	Genes	miRNAs	p-value
mRNA metabolic process	92	6	1.34E-19
cellular protein metabolic process	149	7	3.2E-19
nucleic acid binding transcription factor activity	264	8	3.65E-19
nucleobase-containing compound catabolic process	279	8	4.5E-19
Fc-epsilon receptor signaling pathway	69	8	7.6E-19
cell death	277	9	9.65E-19
neurotrophin TRK receptor signaling pathway	106	9	9.9E-19
membrane organization	209	9	1.201E-18
protein binding transcription factor activity	213	10	2.145E-18
response to stress	609	10	3.456E-18
macromolecular complex assembly	282	10	4.53E-18
mitotic cell cycle	188	11	5.63E-18
catabolic process	607	11	7.6E-18
cellular component assembly	399	11	9.34E-18
small molecule metabolic process	640	11	9.37E-18
enzyme binding	477	13	1.276E-17
symbiosis, encompassing mutualism through parasitism	264	13	2.1E-17
nucleoplasm	504	14	2.18E-17
viral process	234	14	4.89E-17
poly(A) RNA binding	522	14	4.89E-17
biosynthetic process	1311	16	5.437E-17
cytosol	939	17	5.647E-17
cellular protein modification process	816	17	6.54E-17
ion binding	1717	17	6.71E-17
protein complex	1226	17	8.6E-17
RNA binding	678	18	9.82E-17
gene expression	332	19	7.5E-16
cellular nitrogen compound metabolic process	1647	21	9.6E-16
organelle	3270	23	1.8E-15
RNA metabolic process	90	5	1.89E-15
transcription, DNA-templated	555	6	2.29E-14
protein complex assembly	189	4	5.08E-12
DNA metabolic process	196	5	1.25E-11
blood coagulation	118	6	1.31E-09
cytoskeletal protein binding	178	5	2.25E-09
G2/M transition of mitotic cell cycle	53	4	4.28E-08
epidermal growth factor receptor signaling pathway	55	3	6.24E-07
transcription initiation from RNA polymerase II promoter	78	5	9.1E-07
RNA splicing	87	5	1.09E-06
mRNA processing	121	4	4.43E-06
microtubule organizing center	107	3	5.08E-06
enzyme regulator activity	154	3	0.0000402
Fc-gamma receptor signaling pathway involved in phagocytosis	24	3	0.0001053

positive regulation of protein insertion into mitochondrial membrane involved in apoptotic signaling pathway	17	5	0.0001221
immune system process	214	2	0.0001239
fibroblast growth factor receptor signaling pathway	46	2	0.0001266
cell cycle	163	2	0.0002052
chromatin organization	42	3	0.0017787
intrinsic apoptotic signaling pathway	28	4	0.001842
G1/S transition of mitotic cell cycle	53	3	0.002041
transcription factor binding	123	3	0.0030848
nuclear-transcribed mRNA catabolic process, deadenylation-dependent decay	25	2	0.0036671
mRNA splicing, via spliceosome	38	3	0.0059903
vesicle-mediated transport	159	2	0.0073256
innate immune response	128	4	0.0073332
viral life cycle	31	3	0.0161939
cellular lipid metabolic process	41	3	0.022398
positive regulation of transcription, DNA-templated	131	1	0.0319244
cellular component disassembly involved in execution phase of apoptosis	16	2	0.0431022

Supplementary Table 5. Expression difference of top ranked miRNAs across stages of patients with ovarian cancers.

miRNA	Comparison	p-value
hsa-miR-19b-1	Stage 1 vs Stage 2,3&4	N/A
	Stage 2 vs Stage 3	1.635040E-01
	Stage 2 vs Stage 4	1.423760E-01
	Stage 3 vs Stage 4	6.580000E-01
hsa-let-7f-1	Stage 1 vs Stage 2,3&4	N/A
	Stage 2 vs Stage 3	8.590100E-02
	Stage 2 vs Stage 4	2.215400E-01
	Stage 3 vs Stage 4	7.597800E-01
hsa-miR-128	Stage 1 vs Stage 2,3&4	N/A
	Stage 2 vs Stage 3	6.593800E-01
	Stage 2 vs Stage 4	1.600640E-01
	Stage 3 vs Stage 4	9.919900E-02
hsa-miR-1978	Stage 1 vs Stage 2,3&4	N/A
	Stage 2 vs Stage 3	N/A
	Stage 2 vs Stage 4	N/A
	Stage 3 vs Stage 4	N/A
hsa-miR-323a	Stage 1 vs Stage 2,3&4	N/A
	Stage 2 vs Stage 3	6.543400E-01
	Stage 2 vs Stage 4	4.080600E-01
	Stage 3 vs Stage 4	1.367940E-02
hsa-miR-1237	Stage 1 vs Stage 2,3&4	N/A
	Stage 2 vs Stage 3	4.797800E-01
	Stage 2 vs Stage 4	4.962600E-01
	Stage 3 vs Stage 4	9.581400E-01
hsa-miR-486	Stage 1 vs Stage 2,3&4	N/A
	Stage 2 vs Stage 3	4.861000E-01

	Stage 2 vs Stage 4	2.439200E-01
	Stage 3 vs Stage 4	4.852600E-01
hsa-miR-98	Stage 1 vs Stage 2,3&4	N/A
	Stage 2 vs Stage 3	2.358600E-01
	Stage 2 vs Stage 4	4.671800E-01
	Stage 3 vs Stage 4	3.117600E-01
hsa-miR-933	Stage 1 vs Stage 2,3&4	N/A
	Stage 2 vs Stage 3	5.775000E-01
	Stage 2 vs Stage 4	7.479000E-01
	Stage 3 vs Stage 4	N/A
hsa-miR-889	Stage 1 vs Stage 2,3&4	N/A
	Stage 2 vs Stage 3	4.949600E-01
	Stage 2 vs Stage 4	2.817400E-01
	Stage 3 vs Stage 4	6.191800E-02

Supplementary Table 6. Top 10 ranked miRNAs and experimentally validated evidences across various cancer types.

Rank	miRNA	Cancer type	Source
1	hsa-miR-19b	ovarian cancer<br>breast cancer<br>lung cancer	[1–3]
2	hsa-let-7f	ovarian cancer<br>glioma<br>Breast cancer	[4–6]
3	hsa-miR-323	ovarian cancer<br>prostate cancer<br>glioblastoma	[7–9]
4	hsa-miR-1978	endometriosis<br>Breast cancer	[10, 11]
5	hsa-miR-128	ovarian cancer<br>colorectal cancer<br>Gastric cancer	[12–14]
6	hsa-miR-1237	Gastric cancer	[15]
7	hsa-miR-486	ovarian cancer<br>oral cancer<br>gastric Adenocarcinoma	[16–18]
8	hsa-miR-98	ovarian cancer<br>pancreatic ductal adenocarcinoma	[19, 20]
9	hsa-miR-933	Breast cancer	[21]
10	hsa-miR-889	cervical cancer<br>colorectal cancer	[22, 23]

Supplementary Table 7. Prediction list of the identified miRNA signature associated with ovarian cancer based on known associations in dbDEMC 2.0 and HMDD v3.0 databases.

Rank	MiRNA	Cancer type	Expression status	Experiment ID	LogFC
1	hsa-miR-19b	ovarian cancer	up	EXP00327	0.20
2	hsa-let-7f	ovarian cancer	down	EXP00259	-0.93
3	hsa-miR-323	-	-	-	-
4	hsa-miR-1978	-	-	-	-
5	hsa-miR-128	ovarian cancer	up	EXP00260	1.44
6	hsa-miR-1237	ovarian cancer	up	EXP00327	0.48
7	hsa-miR-486	ovarian cancer	up	-	-
8	hsa-miR-98	ovarian cancer	down	EXP00259	-1.07
9	hsa-miR-933	-	-	-	-
10	hsa-miR-889	ovarian cancer	down	EXP00327	-0.41
11	hsa-miR-301b	ovarian cancer	up	EXP00301	1.98
12	hsa-miR-514	ovarian cancer	down	EXP00327	-0.25
13	hsa-miR-935	ovarian cancer	up	EXP00260	0.86
14	hsa-miR-653	ovarian cancer	down	EXP00260	-0.21
15	hsa-miR-1251	ovarian cancer	down	EXP00301	-2.78
16	hsa-miR-616	-	-	-	-
17	hsa-miR-662	ovarian cancer	up	EXP00327	0.49
18	hsa-miR-182	ovarian cancer	up	EXP00259	5.23
19	hsa-miR-1245	-	-	-	-
20	hsa-miR-200c	ovarian cancer	up	EXP00260	4.13
21	hsa-miR-34a	ovarian cancer	down	EXP00260	-0.16
22	hsa-miR-187	ovarian cancer	up	EXP00260	3.92
23	hsa-miR-190	-	-	-	-
24	hsa-miR-342	-	-	-	-
25	hsa-miR-513a	-	-	-	-
26	hsa-miR-146b	ovarian cancer	up	29518404	-
27	hsa-miR-1197	ovarian cancer	down	EXP00327	-0.24
28	hsa-miR-577	-	-	-	-
29	hsa-miR-185	ovarian cancer	down	23318422	-
30	hsa-miR-212	ovarian cancer	down	EXP00260	-1.71
31	hsa-miR-874	ovarian cancer	down	EXP00260	-2.11
32	hsa-miR-1304	ovarian cancer	up	EXP00327	0.25
33	hsa-miR-106b	ovarian cancer	up	EXP00260	1.55
34	hsa-miR-31	ovarian cancer	up	EXP00327	0.26
35	hsa-miR-320d	ovarian cancer	down	EXP00259	-0.81
36	hsa-miR-1295	-	-	-	-
37	hsa-miR-664	ovarian cancer	up	EXP00260	1.09
38	hsa-let-7b	ovarian cancer	down	EXP00327	-0.29
39	hsa-miR-24	ovarian cancer	up	EXP00260	0.55

Supplementary Table 8. Clinical information of independent test cohort.

Race	Asian	11
	black or african american	5
	NA	8
	White	136
Age in years (average)	56.76	
Cancer stage	Stage I	1
	Stage II	10
	Stage III	127
	Stage IV	22
Ethnicity	hispanic or latino	3
	Not hispanic or latino	101
	NA	56
Follow-up time in days (average)	454.27	

REFERENCES

1. Liu M, Yang R, Urrehman U, Ye C, Yan X, Cui S, Hong Y, Gu Y, Liu Y, Zhao C, Yan L, Zhang CY, Liang H, Chen X. MiR-19b suppresses PTPRG to promote breast tumorigenesis. Oncotarget. 2016; 7:64100–08. https://doi.org/10.18632/oncotarget.11799 PMID:27602768

2. Penyige A, Márton É, Soltész B, Szilágyi-Bónizs M, Póka R, Lukács J, Széles L, Nagy B. Circulating miRNA Profiling in Plasma Samples of Ovarian Cancer Patients. Int J Mol Sci. 2019; 20:4533. https://doi.org/10.3390/ijms20184533 PMID:31540229

3. Bulgakova O, Zhabayeva D, Kussainova A, Pulliero A, Izzotti A, Bersimbaev R. miR-19 in blood plasma reflects lung cancer occurrence but is not specifically associated with radon exposure. Oncol Lett. 2018; 15:8816–24. https://doi.org/10.3892/ol.2018.8392 PMID:29805621

4. White NM, Chow TF, Mejia-Guerrero S, Diamandis M, Rofael Y, Faragalla H, Mankaruous M, Gabril M, Girgis A, Yousef GM. Three dysregulated miRNAs control kallikrein 10 expression and cell proliferation in ovarian cancer. Br J Cancer. 2010; 102:1244–53. https://doi.org/10.1038/sj.bjc.6605634 PMID:20354523

5. Yan S, Han X, Xue H, Zhang P, Guo X, Li T, Guo X, Yuan G, Deng L, Li G. Let-7f Inhibits Glioma Cell Proliferation, Migration, and Invasion by Targeting Periostin. J Cell Biochem. 2015; 116:1680–92. https://doi.org/10.1002/jcb.25128 PMID:25735962

6. Elghoroury EA, ElDine HG, Kamel SA, Abdelrahman AH, Mohammed A, Kamel MM, Ibrahim MH. Evaluation of miRNA-21 and miRNA Let-7 as Prognostic Markers in Patients With Breast Cancer. Clin Breast Cancer. 2018; 18:e721–26. https://doi.org/10.1016/j.clbc.2017.11.022 PMID:29292183

7. Vaksman O, Tropé C, Davidson B, Reich R. Exosome-derived miRNAs and ovarian carcinoma progression. Carcinogenesis. 2014; 35:2113–20. https://doi.org/10.1093/carcin/bgu130 PMID:24925027

8. Gao Q, Yao X, Zheng J. MiR-323 Inhibits Prostate Cancer Vascularization Through Adiponectin Receptor. Cell Physiol Biochem. 2015; 36:1491–98. https://doi.org/10.1159/000430313 PMID:26160610

9. Shahar T, Granit A, Zrihan D, Canello T, Charbit H, Einstein O, Rozovski U, Elgavish S, Ram Z, Siegal T, Lavon I. Expression level of miRNAs on chromosome 14q32.31 region correlates with tumor aggressiveness and survival of glioblastoma patients. J Neurooncol. 2016; 130:413–22. https://doi.org/10.1007/s11060-016-2248-0 PMID:27573219

10. Suryawanshi S, Vlad AM, Lin HM, Mantia-Smaldone G, Laskey R, Lee M, Lin Y, Donnellan N, Klein-Patel M, Lee T, Mansuria S, Elishaev E, Budiu R, et al. Plasma microRNAs as novel biomarkers for endometriosis and endometriosis-associated ovarian cancer. Clin Cancer Res. 2013; 19:1213–24. https://doi.org/10.1158/1078-0432.CCR-12-2726 PMID:23362326

11. Fix LN, Shah M, Efferth T, Farwell MA, Zhang B. MicroRNA expression profile of MCF-7 human breast

cancer cells and the effect of green tea polyphenon-60. Cancer Genomics Proteomics. 2010; 7:261–77. PMID:20952761

12. Li B, Chen H, Wu N, Zhang WJ, Shang LX. Deregulation of miR-128 in ovarian cancer promotes cisplatin resistance. Int J Gynecol Cancer. 2014; 24:1381–88. https://doi.org/10.1097/IGC.0000000000000252 PMID:25248111

13. Wu L, Shi B, Huang K, Fan G. MicroRNA-128 suppresses cell growth and metastasis in colorectal carcinoma by targeting IRS1. Oncol Rep. 2015; 34:2797–805. https://doi.org/10.3892/or.2015.4251 PMID:26352220

14. Liu HT, Xing AY, Chen X, Ma RR, Wang YW, Shi DB, Zhang H, Li P, Chen HF, Li YH, Gao P. MicroRNA-27b, microRNA-101 and microRNA-128 inhibit angiogenesis by down-regulating vascular endothelial growth factor C expression in gastric cancers. Oncotarget. 2015; 6:37458–70. https://doi.org/10.18632/oncotarget.6059 PMID:26460960

15. Liu J, Ma L, Wang Z, Wang L, Liu C, Chen R, Zhang J. MicroRNA expression profile of gastric cancer stem cells in the MKN-45 cancer cell line. Acta Biochim Biophys Sin (Shanghai). 2014; 46:92–99. https://doi.org/10.1093/abbs/gmt135 PMID:24384510

16. Nakamura N, Terai Y, Nunode M, Kokunai K, Konishi H, Taga S, Nakamura M, Yoo M, Hayashi M, Yamashita Y, Ohmichi M. The differential expression of miRNAs between ovarian endometrioma and endometriosis-associated ovarian cancer. J Ovarian Res. 2020; 13:51. https://doi.org/10.1186/s13048-020-00652-5 PMID:32359364

17. Chou ST, Peng HY, Mo KC, Hsu YM, Wu GH, Hsiao JR, Lin SF, Wang HD, Shiah SG. MicroRNA-486-3p functions as a tumor suppressor in oral cancer by targeting DDR1. J Exp Clin Cancer Res. 2019; 38:281. https://doi.org/10.1186/s13046-019-1283-z PMID:31253192

18. Chen H, Ren C, Han C, Wang D, Chen Y, Fu D. Expression and prognostic value of miR-486-5p in patients with gastric adenocarcinoma. PLoS One. 2015; 10:e0119384. https://doi.org/10.1371/journal.pone.0119384 PMID:25793394

19. Wang Y, Bao W, Liu Y, Wang S, Xu S, Li X, Li Y, Wu S. miR-98-5p contributes to cisplatin resistance in epithelial ovarian cancer by suppressing miR-152 biogenesis via targeting Dicer1. Cell Death Dis. 2018; 9:447. https://doi.org/10.1038/s41419-018-0390-7 PMID:29670086

20. Fu Y, Liu X, Chen Q, Liu T, Lu C, Yu J, Miao Y, Wei J. Downregulated miR-98-5p promotes PDAC proliferation and metastasis by reversely regulating MAP4K4. J Exp Clin Cancer Res. 2018; 37:130. https://doi.org/10.1186/s13046-018-0807-2 PMID:29970191

21. Tanman Ü, Yangın S, Cansaran-Duman D. Determination of Dysregulated miRNA Expression Levels by qRT-PCR after the Application of Usnic Acid to Breast Cancer. Anticancer Agents Med Chem. 2020; 20:548–58. https://doi.org/10.2174/1871520619666190923163552 PMID:31549595

22. Sun Y, Cheng Y, Zhang Y, Han K. MicroRNA-889-3p targets FGFR2 to inhibit cervical cancer cell viability and invasion. Exp Ther Med. 2019; 18:1440–48. https://doi.org/10.3892/etm.2019.7675 PMID:31316631

23. Xiao Y, Li ZH, Bi YH. MicroRNA-889 promotes cell proliferation in colorectal cancer by targeting DAB2IP. Eur Rev Med Pharmacol Sci. 2019; 23:3326–34. https://doi.org/10.26355/eurrev_201904_17695 PMID:31081086
